# The design, implementation and acceptability of an integrated intervention to address multiple behavioral and psychosocial risk factors among pregnant African American women

**DOI:** 10.1186/1471-2393-8-22

**Published:** 2008-06-25

**Authors:** Kathy S Katz, Susan M Blake, Renee A Milligan, Phyllis W Sharps, Davene B White, Margaret F Rodan, Maryann Rossi, Kennan B Murray

**Affiliations:** 1 Department of Pediatrics, Georgetown University Medical Center, 2201 Wisconsin Ave NW, Suite 220, Washington DC 20007, USA; 2 School of Public Health and Health Services, George Washington University, 2175 K St. NW, Suite 700, Washington, DC 20037, USA; 3 Johns Hopkins University School of Nursing, 525 N. Wolfe St., Baltimore, MD 21205, USA; 4 Department of Pediatrics, Howard University Hospital, 2041 Georgia Ave NW, Washington DC 20060, USA; 5 Office for the Protection of Human Subjects, Children's Hospital National Medical Center, 111 Michigan Avenue, NW, Washington, DC 20010, USA; 6 Research Triangle Institute-International, 6110 Executive Blvd, Rockville MD 20850, USA

## Abstract

**Background:**

African American women are at increased risk for poor pregnancy outcomes compared to other racial-ethnic groups. Single or multiple psychosocial and behavioral factors may contribute to this risk. Most interventions focus on singular risks. This paper describes the design, implementation, challenges faced, and acceptability of a behavioral counseling intervention for low income, pregnant African American women which integrated multiple targeted risks into a multi-component format.

**Methods:**

Six academic institutions in Washington, DC collaborated in the development of a community-wide, primary care research study, DC-HOPE, to improve pregnancy outcomes. Cigarette smoking, environmental tobacco smoke exposure, depression and intimate partner violence were the four risks targeted because of their adverse impact on pregnancy. Evidence-based models for addressing each risk were adapted and integrated into a multiple risk behavior intervention format. Pregnant women attending six urban prenatal clinics were screened for eligibility and risks and randomized to intervention or usual care. The 10-session intervention was delivered in conjunction with prenatal and postpartum care visits. Descriptive statistics on risk factor distributions, intervention attendance and length (i.e., with < 4 sessions considered minimal adherence) for all enrolled women (n = 1,044), and perceptions of study participation from a sub-sample of those enrolled (n = 152) are reported.

**Results:**

Forty-eight percent of women screened were eligible based on presence of targeted risks, 76% of those eligible were enrolled, and 79% of those enrolled were retained postpartum. Most women reported a single risk factor (61%); 39% had multiple risks. Eighty-four percent of intervention women attended at least one session (60% attended ≥ 4 sessions) without disruption of clinic scheduling. Specific risk factor content was delivered as prescribed in 80% or more of the sessions; 78% of sessions were fully completed (where all required risk content was covered). Ninety-three percent of the subsample of intervention women had a positive view of their relationship with their counselor. Most intervention women found the session content helpful. Implementation challenges of addressing multiple risk behaviors are discussed.

**Conclusion:**

While implementation adjustments and flexibility are necessary, multiple risk behavioral interventions can be implemented in a prenatal care setting without significant disruption of services, and with a majority of referred African American women participating in and expressing satisfaction with treatment sessions.

## Background

While infant mortality rates in the US have shown a decrease of nearly 23% in the past decade, significant disparities continue to exist for some racial-ethnic groups, particularly for African-Americans [1]. In Washington DC, with a predominately African American population, the overall infant mortality rate fell from 18.6/1000 live births in 1992 to 11.6/1000 in the year 2003 [2,3]. However, African American infant death rates in DC continued to be nearly three times that of white DC infants and two and a half times that of the US as a whole. [3]. The NIH-DC Initiative to Reduce Infant Mortality in Minority Populations, a congressionally mandated community-based research program, was created to address the high rate of infant mortality and morbidity in Washington, DC. The study described in this manuscript, DC-HOPE, was initiated in 2000.

### The NIH-DC Initiative

The NIH-DC Initiative in Washington, DC, is a collaboration among four academic research institutions (Children's National Medical Center, Georgetown University, George Washington University, Howard University), a data coordinating center (RTI International) and the National Institutes of Health (National Institute of Child Health and Human Development, National Center on Minority Health and Health Disparities). Phase II (1997–2003) of the DC Initiative focused on a multiple risk factor intervention trial, Healthy Outcomes of Pregnancy Education (DC-HOPE), to reduce behavioral and psychosocial risks for adverse infant health outcomes among pregnant minority women in Washington DC. This randomized intervention trial targeted four risk factors with demonstrated associations with preterm delivery, low birth weight, and infant mortality: maternal cigarette smoking, environmental tobacco smoke exposure (ETSE), depression, and intimate partner violence (IPV). Additionally, an educational component addressing reproductive behavioral health risks such as unintended pregnancy and sexually transmitted infections (STIs) was included with any one of these four risks.

Although multiple risk factors are associated with increased morbidity and mortality for many health outcomes, most health promotion interventions tend to apply single rather than multiple risk behavior approaches [4,5]. Multiple risk behavior interventions in communities, primary care and school settings, have primarily focused on the prevention of cardiovascular and cancer disease risks [6-9]. Such interventions may result in a complexity of design that can make it difficult to achieve full integration. While interventions that focus on multiple factors contributing to health outcomes may be more effective, they can also increase participant burden by emphasizing change in several behaviors at once [5]. Although interventions to prevent poor pregnancy outcomes may have significant population benefits, few multiple risk factor interventions have been designed or tested for efficacy in prenatal care settings.

Evidence points to the importance of addressing interactions between lifestyle behaviors, the social environment, and health outcomes. Conclusions drawn from reviews of primary care interventions highlight the following: 1) behavioral counseling interventions are underutilized in healthcare settings [10], 2) behavioral counseling interventions in primary care settings may help people change when risk behaviors are linked [10], 3) intervention strategies should focus on ways to facilitate adoption of implementation practices into routine health care [11], and 4) addressing multiple risk factors simultaneously versus sequentially may be more effective [6].

The purpose of this paper is to describe the conceptual design, implementation characteristics, and acceptability of a multiple risk factor intervention. This paper provides a full report of the feasibility of implementing psychosocial and behavioral interventions in prenatal care settings, to address single or multiple risks presented by inner city African American women, which place them at risk for poor pregnancy outcomes. For health professionals considering offering psychosocial and behavioral interventions in primary health care settings, a summary of challenges faced in the DC-HOPE study is also provided. In order to give proper attention to these issues, the effects of the intervention on pregnancy outcome and risk reduction will be described in separate papers.

## Methods

### Conceptual Framework for Integrated Intervention Development

**Figure 1:**
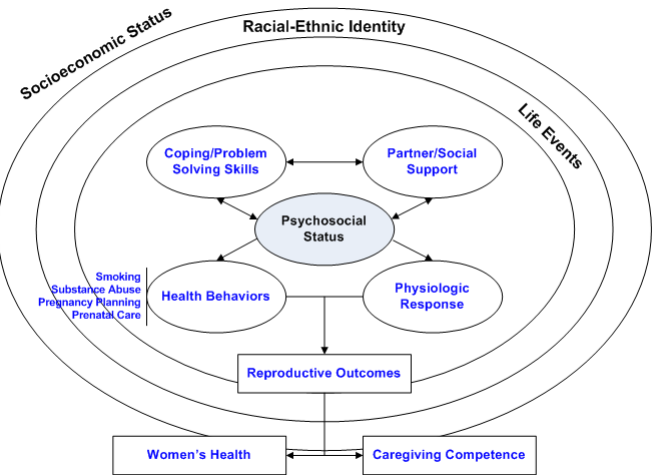
Schema for DC-HOPE Conceptual Framework.

This integrated intervention was built on a conceptual framework that posits the interactive role of the individual and the social environment, with overarching contextual factors which may influence psychosocial stresses, health behaviors and pregnancy outcomes. African American minority racial status and low income socioeconomic status were considered as overarching contextual variables that may be associated with daily stresses and major life events, and which can significantly impact psychosocial status (See Figure 1). The presence of positive social supports can serve to mediate the impact of these stressful life events on psychosocial status and facilitate positive emotional coping and problem solving skills. Absent support or negative social support (e.g. an abusive partner) may make a woman more vulnerable to poor psychological outcomes such as depression. Psychosocial status can also impact health behaviors, which can in turn affect pregnancy outcomes and child morbidity. These behaviors may have both positive and negative consequences and include such things as use of alcohol, drugs and tobacco, and contraceptive use. Psychosocial stresses may contribute to adverse health care behaviors such as delayed prenatal care initiation, inadequate attention to reproductive behavioral health issues, and can impact physiological responses as well, furthering the risks for poor infant health outcomes.

As proposed, this framework attempts to address multiple levels of influence and includes some, but not all, of the constructs found in social ecological models of behavior change [12,13]. The DC-HOPE intervention combined elements from social ecological, transtheoretical and cognitive behavioral treatment models to address multiple existing risks simultaneously. Risk interventions all addressed negative aspects of relationships that contributed to risk status and how to identify or develop supportive ones. All intervention components had elements addressing coping and problem solving skills. Finally, interventions for smoking and depressive symptoms used cognitive behavioral strategies to revise perceptions that sustained the woman's risk status. For many women, these psychosocial and behavioral risks may overlap, and multiple risks may be present in an individual during pregnancy [14]. By intervening on a single risk factor, treatment might be unsuccessful because other risks continue to serve as barriers to the desired change [15].

### Development of the Risk Specific Intervention Components

#### Tobacco Smoke Product Exposure

##### Cigarette Smoking

The adverse effects of tobacco smoke exposure on infant health outcomes are dose-dependent for both active [16] and passive smoke exposure [17,18]. Cigarette smoking during pregnancy adversely affects reproductive health outcomes including intrauterine growth retardation, small-for-gestational-age, preterm birth, stillbirth, spontaneous abortion, and placental abruption [19-21]. Women who quit smoking early in pregnancy have infants with birth weights close to non-smoking mothers [16,22]. However, benefits can occur even when women quit smoking in their third trimester [23,24].

One of the most successful interventions for pregnant smokers was the Smoking Cessation or Reduction in Pregnancy Program Treatment (SCRIPT) trial. Based on Social Cognitive Theory [25], the SCRIPT trial increased quit rates from 2% to 20% and significant reduction rates from 7% to 30% [26-29]. Other studies have applied the Transtheoretical Model [30] to interventions with pregnant women, with its emphasis on tailoring advice to a woman's stage of readiness for behavior change [31] often through the use of motivational interviewing [15,32]. Some studies found no differences in quit rates or reduction [32,33], whereas others found small to modest effects [34-37].

Cognitive Behavioral Therapy (CBT) interventions have gained widespread application in the treatment of substance abuse, and smoking in particular, but have been less frequently reported among pregnant populations [38-40]. Abstinence rates following group-based CBT interventions with non-pregnant adult smokers ranged from 33–69% [41-43], whereas cessation rates reported among pregnant women were generally lower.

The DC-HOPE smoking intervention component combined elements from several of these theoretical models and treatment approaches. In keeping with the conceptual framework of the study, the intervention was designed to address positive and negative aspects of social relationships, negative cognitions and positive coping strategies to encourage cessation or reduction of risk. Women who currently or had recently smoked (within 6 months of becoming pregnant) were assigned to the active smoking intervention. This intervention included content addressing both active smoking and smoke exposure, whether or not the women also met criteria for environmental tobacco smoke exposure (ETSE). Consistent with SCRIPT and The Counseling and Behavioral Interventions Work Group of the United States Preventive Services Task Force (USPSTF) recommendations, a five-step behavioral counseling approach (The Five A's) was used to include: 1) Ask about smoking status, 2) Advise to quit, 3) Assess willingness to quit, 4) Assist in ways to quit, and 5) Arrange for follow-up [10,44]. Adapted SCRIPT materials were presented at the first visit, and included viewing a 6-minute version of the "Commit to Quit" videotape, and giving women "A Pregnant Woman's Guide to Quit Smoking" for home use. At each intervention visit, assessments were made of current stage of change and strategies used to quit or reduce smoking or avoid ETSE since the last visit. Smoking cessation content thereafter was tailored to a woman's progress along the continuum of change using a collapsed version of the five stages of change [31]: 1) Precontemplation + contemplation; 2) Preparation; and 3) Action + maintenance. CBT approaches were used, along with motivational interviewing [36,40], to encourage women to consider how their thoughts and feelings affect behavior and to promote progression across stages. Participants were also encouraged to self-monitor smoking habits and identify common smoking triggers, including ETSE. Once identified, women were encouraged to avoid triggers and educated in the use of alternative coping and behavioral change strategies. Counselors provided reinforcement for successive changes toward smoke avoidance and smoking cessation/reduction, as well as skills practice and role plays for negotiation with partners and household members who smoked.

##### Environmental Tobacco Smoke Exposure (ETSE)

Adverse effects of prenatal ETSE include low birth weight and other negative health outcomes [17,21,45-52]. Evidence for the adverse effects of prenatal ETSE has been most consistent in relation to birth weight [17,21,45-50], but there is some support for associations between higher ETSE levels and preterm delivery, small-for-gestational age babies, and fetal death [17,21,45,51,52]. Consistent with studies of cigarette smoking [53-56] adverse effects of ETSE were higher among African American women [21].

Few studies have focused on prevention of ETSE during pregnancy, despite evidence that women, especially recently quitters, continue to be exposed to smoke by partners, family members and friends [33,57]. The ETSE intervention component, addressing passive smoke exposure, used in this study paralleled the active smoking content both in terms of theoretical concepts and prevention strategies. Whereas current or recent smokers received the smoking intervention (which included content addressing both active smoking and passive smoke exposure), non-smokers who were exposed to smoke received a specific ETSE intervention. The focus for women who reported ETSE during pregnancy included 1) ETSE avoidance and reduction, and 2) changing the surrounding environment through negotiation skills and creating household smoking bans. All women randomized to the intervention group, irrespective of risk status, received information at their first postpartum session about the risks of ETSE for their newborn infants, and how to protect their infants from harm.

#### Depression

A number of studies have found associations between depression and reproductive outcomes. High levels of depressive symptoms have been found to be significantly associated with preterm delivery among low-income African American women [58] and among a low income rural population [59]. Steer et al [60] found a direct relationship between increases in scores for depressive symptoms on the Beck Depression Inventory and poor pregnancy outcomes among African American and Latina adults; clinically depressed women in this sample had a threefold increase for prematurity or low birth weight. Negative mood, unhappiness about the pregnancy, maternal smoking and low maternal weight gain have also been associated with increases in low birth weight rates [61]. Among pregnant women living in poverty, there is a high incidence of depressive symptoms [62]. Prevalence of depressive symptoms in low-income pregnant and parenting women ranges from 20–50% when identified by self-report screening tools [63], but when combined with diagnostic interview for major or minor depression yields a more conservative estimate of 8.5 to 11% [64]. Furthermore, negative health behaviors have often been associated with depression in women. Attempts to ward off depressive symptoms through "self-medication" may lead women to use tobacco, drugs or alcohol, or engage in negative health behaviors which contribute to poor pregnancy outcomes [65]. Most interventions for depression in pregnant women have focused on prevention of postpartum depression. A few interventions have been conducted in prenatal care settings to treat perinatal depression. Most of these studies screened and enrolled women at risk during the perinatal period and followed them over several months postpartum. Intervention approaches and success rates varied, with some studies demonstrating treatment versus usual care differences postpartum [66-68], but the majority of others did not [69-72]. Several of the above intervention studies applied CBT approaches [66,69,71], two tested an Interpersonal Therapy model [67,68], and at least one highlighted the potential importance of taking an individual vs. group-based approach to CBT [71]. However, it was unclear how the three successful interventions [66-68] differed from the others, one implemented 5–8 individual CBT counseling sessions in women's homes, whereas the other two, with very small samples, tested group-based interpersonal therapy interventions. Depressive symptoms seem to abate during the course of pregnancy for most women, irrespective of intervention, making it difficult to determine specific treatment effects [70,73].

Past studies have described the complexity of implementing psychotherapeutic approaches to depression treatment in primary care particularly with minority populations [74]. Continued disparities in access to mental health care for minorities and some socioeconomic groups, supports the importance of testing the feasibility of such interventions in primary care settings [75]. A group-based CBT intervention developed by Miranda and Munoz [76] was successful with depressed primary care patients and with a low-income, ethnic minority obstetric/gynecological population.

This treatment model represents a good fit with the conceptual framework of DC-HOPE in addressing negative cognitions and relationships that contribute to depressive symptoms in minority women. The syllabus, treatment manual and support materials developed as part of the Miranda and Munoz [76] treatment model were adapted for use in the DC-HOPE intervention. The original group therapy model was adapted in DC-HOPE to an individual treatment format that consisted of an 8-session sequence delivered at the clinic in conjunction with prenatal visits. The prenatal care visit intervention sessions focused on secondary prevention of depressive symptoms during pregnancy, and two postnatal booster sessions addressed possible increased depression vulnerability in the postpartum period. CBT strategies for mood management, increasing pleasurable activities, and increasing positive social interactions were the major focus of the depression intervention. Each session focused on skill development of cognitive strategies for revising negative cognitions. Homework assignments, such as planning and carrying out a pleasurable activity, were designed to allow for skill practice in the woman's real-life settings between intervention sessions.

#### Intimate Partner Violence

It is estimated that 2 million women, annually, are the victims of physical, emotional and sexual violence by an intimate partner such as a husband, ex-husband, boyfriend, or ex-boyfriend [77,78]. Among African American women between the ages of 15–24 years, IPV is a leading cause of death and non-lethal injury [79]. Three to 19% of pregnant women experience adverse pregnancy outcomes either for themselves or their unborn infants attributable to IPV [80]. Maternal consequences include traumatic injury, miscarriage, late entry into prenatal care, poor weight gain, and increased risk for STIs [81,82]. Neonatal consequences include fetal injury, low birth weight, and preterm delivery [83-87]. Mental health problems, particularly depression, may overlap with intimate partner violence for women [88]. Behavioral health problems associated with IPV for women include use of drugs, alcohol and cigarettes [84,89,90] which increase risks for poor pregnancy outcomes [91].

Despite evidence for adverse infant health outcomes, few IPV prevention interventions have been conducted during pregnancy [92]. IPV interventions have, however, been designed to be delivered in other medical settings, such as the structured IPV intervention developed by Parker and colleagues [80], which was based upon Dutton's Empowerment theory [93]. This brochure-based intervention includes assessment strategies for health care providers, information for women about IPV, and options for her development of a personal safety plan. Women presented with the Parker intervention reported the adoption of significantly more safety behaviors at 6 and 12 months postpartum as compared to women provided only with a listing of community resources [80].

Parker's model was selected for the Project DC-HOPE IPV component. The model particularly addresses the role of negative partner support as formulated in the conceptual framework of DC-HOPE. The brochure, which was adapted for DC-HOPE, provided information about the types (e.g., emotional, physical and sexual) and the cycle of violence (e.g., escalating, IPV, honeymoon period), a Danger Assessment to identify risks for harm, and preventive options women might consider (e.g., leaving her partner, filing a civil protection order), and the development of a safety plan (e.g., leaving keys or paper documents with others). Lists of community resources which included the addresses and phone numbers for IPV services were provided to all intervention group women. Counselors discussed the components of the brochure with each woman at risk. The approach allowed an individualized focus at each intervention visit on areas of particular need for that woman. While the Parker model was designed for delivery in one visit, the Project DC-HOPE adaptation enabled monitoring each woman's progress over a 10-session sequence in developing a safety plan and considering options to remaining with her partner.

#### Reproductive Behavioral Health Risks

Relationships among unintended pregnancies, sexually transmitted infections (STIs), and infant morbidity/mortality are compelling. Risky behaviors such as unprotected sex or having multiple sexual partners increase the chance of acquiring STIs and unintended pregnancies [94,95]. Unprotected sexual intercourse is associated with unintended pregnancy, short interpartum intervals and STIs, all of which can result in poor perinatal outcomes [96-98].

Because of the empirical support for the relationship of pregnancy planning and STIs on reproductive outcomes, the DC-HOPE intervention included a brief, informational prevention component which was provided to all intervention participants, irrespective of a woman's risk status at each of the 10-session visits. All intervention women received basic information about reproductive tract anatomy and physiology, how STIs are spread, the risk of STIs during pregnancy, pregnancy and STI prevention options, and days for pregnancy risk during the menstrual cycle. Women were encouraged to discuss with their doctors those strategies that would work best for them in preventing a subsequent unplanned pregnancy.

### Format of the Integrated, Multiple Risk Factor Intervention

#### Prenatal Session Characteristics

The original models for the risk-specific components of the intervention varied as to the number of sessions required for delivery of the content. The complete depression intervention sequence was originally the longest, involving 8 prenatal sessions and 2 postpartum sessions. The other risk intervention components: smoking, ETSE, and IPV, thus were modified so they could be similarly delivered over a 10-session sequence. Women identified as having a given risk received content related to that risk factor at each session. Four prenatal sessions were hypothetically considered as the minimum number of sessions for exposure to the intervention content that might produce any effect.

A counselor, called a Pregnancy Advisor (PA), sought to keep the overall session length to 45 minutes maximum. Discussion of any particular risk factor in the integrated counseling series was allotted no more than 30 minutes. The content of other risk factor discussions was adjusted accordingly so that the total session time remained within 45 minutes. This was not a problem for women with one or two risks, but did present some difficulties for women with all 3 risks. To address this issue for women with all 3 risks, the counselor varied the time spent on a particular risk in any given session, so that all risks would eventually be fully addressed over time.

All PAs followed a standardized sequence at each session for the order in which information was presented to women with multiple risks. Cigarette smoking and/or ETSE, the risk with the greatest prevalence in the study group, was presented first. When indicated, depression was the next topic in the sequence followed by IPV for those at risk. The reproductive behavioral health component which was presented to all women, regardless of other risks, was a concise and focused discussion during the closing few minutes of each session. The sequence of risk presentation was adjusted, as necessary, to address any critical issues that the woman reported.

Homework assignments called "take home tasks" were included as part of each risk component. These were designed to carry over treatment goals between sessions. For smoking, these might include keeping track of the time and situation in which the woman smoked over a several day period. For IPV it might be taking steps in a safety plan such as discussing a code word with a trusted neighbor that would indicate that the woman was in danger and needed help. For depression, setting a specific time for an activity that brought the woman pleasure might be the assignment. These tasks were negotiated with the woman in keeping with what she thought she could do and provided as a written reminder at the conclusion of the session.

Bulleted visual text, supporting the issues targeted during the session, was presented to the woman on a computer screen. The visual support materials were developed in Microsoft PowerPoint format. A menu of the targeted risk factors, and hyperlinks for specific session numbers on the computer allowed the PA to quickly access the materials for the designated session. The PA also had a treatment manual to guide her presentation of the content of each session. The woman's active participation was encouraged, but the PA tactfully limited non-curriculum based discussion.

The intervention occurred concurrently with prenatal care visits, to reduce time burden and maximize participation. The intervention office space was contiguous with regular prenatal care clinic space and efforts were made to minimize any disruptions to the clinic flow. Intervention sessions were delivered during a woman's wait for her medical examination or following her visit with healthcare providers.

If a woman missed her prenatal clinic visit, the PA attempted to contact the woman by phone to indicate her hope that the woman would reschedule the clinic visit to enable them to meet together for an intervention session.

#### Postpartum sessions

A maximum of two intervention sessions were provided in the postpartum period within 8 weeks of delivery. These sessions were designed to reinforce risk specific intervention goals and support women through the postpartum period. Irrespective of reported risk, each woman was guided in filling gaps in her support network. Her plan for maintaining good reproductive health was reviewed, barriers identified, and solutions discussed. Smoking risk, ETSE, depression and/or intimate partner violence intervention goals were updated in keeping with the postpartum environment. At the first postpartum visit, strategies to prevent ETSE for the newborn infant were discussed with all women.

### The Counseling staff

Pregnancy Advisors (PAs) were hired to deliver the intervention over the course of the study. The majority of the PAs had Masters Degrees in counseling disciplines (e.g., community health, marriage and family, or psychology) and experience in interpersonal counseling, health education or behavior change; two PAs were RNs. This level of experience was considered necessary for application of CBT approaches. All PAs, but two, were African American or Hispanic and had experience in counseling minority inner city populations. All PAs received three weeks of intensive training focused on the background issues (e.g., epidemiology of infant health risks), intervention rationale (e.g., theoretical models and evidence for prior intervention effectiveness), and protocols for the delivery and content for each risk factor and intervention session. The PAs additionally received training from a variety of research collaborative team members with expertise in cultural competence, suicide risk, and motivational interviewing techniques. An intervention training manual with support materials was designed to help prepare the PA for her performance during intervention delivery.

Weekly supervision sessions with a licensed clinical psychologist and input from the risk specialists on the research team were provided to PAs. Particular obstacles with patients – and sometimes with clinic staff – were addressed at these meetings. Periodic direct observations and review of audiotaped transcripts of the PA's intervention sessions were made by the supervisory team. Feedback was provided to the PAs on enhancing patient rapport and presentation style, and adhering with fidelity to the content in the intervention manual.

A mental health consultation team of psychiatrists and clinical psychologists was on call to respond to concerns of the PAs or telephone interviewers about acute mental health status issues, especially suicidal ideation, of study participants.

### Recruitment and Retention of Subjects

Participants were recruited at six prenatal care clinics in Washington, D.C from July 2001 to October 2003. Three were hospital-based prenatal care clinics serving low income women, two were university-based medical center prenatal clinics serving a somewhat broader socioeconomic range of minority women, and one was a Medicaid managed-care clinic serving primarily African American women [98]. Institutional review boards at Howard University, RTI International, and NIH approved the study.

Several strategies were implemented in Project DC-HOPE to promote successful recruitment and retention of study participants [99]. These strategies included features of the study design which included delivery of the interventions during regularly scheduled clinic visits, consistent contact with study participants, financial incentives, recruitment training, cooperation from clinic staff, computerized tracking of study participants, and continuous monitoring of study progress [99]. While the study team recognized the need for empirical research comparing the effectiveness of different approaches to recruitment and retention, as well as the importance of examining factors and methods most sensitive to the needs of ethnic and racial minorities [100], no particular theoretical models were used for recruitment and retention, nor were alternative recruitment or retention strategies tried and tested for efficacy. Instead, practical methods demonstrated to be important in community-based research including reliance on and respect for the clinic infrastructure and staff, hiring culturally sensitive staff, and maintaining rapport and sensitivity to participant experiences were used.

At the prenatal clinic visit, recruitment specialists for the study explained the project to pregnant women who, after providing written consent, were asked to complete a brief self-administered computerized screening battery (Audio-Computer Assisted Self Interview: A-CASI) to assess their eligibility and risk status. In this technology, respondents listen to recorded questions through headphones which are simultaneously displayed on a laptop computer screen. Respondents touch the screen to choose a response option. Women who were identified by ACASI as having smoking, depressive symptoms, or IPV risk were invited to participate in the study. A total of 2,913 women completed the screening, and 1,398 (48%) were eligible and met one or more of the criteria for the targeted risk factors. Of these, 1,070 (76%) women were successfully enrolled in DC-HOPE. Eligibility included: self-identification as Black/African-American or Latina, residence in the District of Columbia, ≥ 18 years of age, English speaking, receiving prenatal care at one of the participating clinics, enrollment by 28 weeks gestation, and reporting one or more of the designated psychosocial and behavioral risk factors for poor pregnancy outcomes.

Eligible women were then consented to participate in the study and completed the baseline questionnaire by telephone interview. After completion of this telephone interview, women were randomized to receive either the integrated intervention or usual care. Site and risk-specific block randomization of participants was conducted.

### Measures

#### A-CASI Screening measure

The A-CASI was designed to identify women reporting risks targeted for intervention. Questions were drawn from previously validated screening measures. Criteria for smoking risk included having smoked within the 6 months before or since becoming pregnant, or any ETSE during pregnancy, as determined by items adapted from the Smoke-Free Families (SFF) core screening questions to assess smoking and ETSE in pregnant and parenting women [101]. Depressive symptoms were screened using the BDI-FastScreen for Medical Patients, a 7-item reduced version of the widely used Beck Depression Inventory, with a score ≥4 meeting criteria for risk [102]. A cut-off of ≥4 was found to yield 98% maximum clinical efficiency with 97% sensitivity and 99% specificity among outpatients when screening for major depression [103]. IPV was identified by the Abuse Assessment Screen (AAS), a measure designed and validated for use in pregnancy [104], if a woman reported physical or sexual abuse by a partner in the previous year.

#### Main Study Assessments

While data on intervention impacts and outcomes will not be presented in this paper, a brief description may be helpful to grounding results from assessments presented in this paper to the larger study context.

During the full study, intervention impacts and outcomes were assessed via telephone interviews, biomarker assessments, and medical record abstractions. Interviews were conducted at baseline (prior to randomization). Participant demographic and background characteristics were obtained on the baseline telephone interview and a battery of baseline measures associated with risk were administered. Measures included were smoking and ETSE abstinence items from the Smoke-Free Families (SFF) core questionnaires [101], the 20-item Hopkins Symptom Checklist-Depression Scale (HSCL-D), a depression symptom screening tool widely used in research studies of depression treatment in primary care [105,106], and the frequency of physical assault and sexual coercion (partner to self), assessing IPV as measured by the Conflict Tactics Scale (CTS) [107,108]. Mediating variables such as self-efficacy, coping, and social and environmental support, and possible confounds such as substance use, were also assessed during the structured telephone interviews.

Depending on a woman's week of gestation at enrollment, either one or two prenatal follow-up interviews were completed during the second and third trimester and one postpartum follow-up interview was completed 6–10 weeks following delivery. All interviews were completed by telephone. Additionally, saliva cotinine samples were collected on a schedule that paralleled the telephone interviews to validate self-reported cigarette smoking and ETSE. Infant morbidity/mortality related data (e.g., prematurity and low birth weight) were collected through infant and maternal medical records abstractions, as well as through maternal self-report during the telephone interviews.

### Intervention Participation and Implementation

The Data Coordinating Center facilitated the collection and monitoring of process evaluation measures through a computerized and centralized data monitoring system [99]. Recruitment, screening, enrollment, and retention statistics, and participant contact information were updated regularly. The PAs reported on delivery of core intervention content and the time spent on each risk factor at each intervention session in order to assess treatment fidelity and adherence to intervention protocols, as well as facilitate the interpretation of findings associated with treatment intensity and dosage in relation to treatment outcome. Such information was deemed necessary to determine whether the interventions were being delivered as intended and prescribed, and to ensure treatment fidelity [109,110]s. Completion of all study assessments were similarly monitored once women were enrolled (e.g., telephone interviews, saliva cotinine, and medical records abstractions). The Data Coordinating Center analyzed these data and provided periodic reports to investigators to monitor study progress. Portions of these data are reported in this paper.

### Participant Satisfaction and Intervention Acceptability

A telephone debriefing interview was also conducted with a 20% random subsample of study participants within 2–4 months following study completion (i.e., completion of the postpartum intervention and interview) to assess their perceptions of and satisfaction with study participation, as well as the acceptability of the intervention. This proportion was deemed sufficient to represent the larger sample, was not overly burdensome, and yielded 78 women in the intervention and 74 women in the usual care group who might offer some perspectives as to how the overall study and the intervention were perceived. All women (both intervention and usual care) who completed the debriefing interviews were asked about their perceptions of study participation and prenatal care in general. Questions for both intervention and control women focused on their perceptions of: 1) the information and skills received during general prenatal care; 2) the information they received about the study prior to enrollment; 3) their experiences with study staff members and procedures at recruitment and during the study (e.g., recruitment, interviews, saliva assessments; and 4) their satisfaction with their overall study participation and prenatal care services in general. Women assigned to the intervention group were additionally asked questions to assess their perceptions of: 1) intervention content, materials and homework assignments; 2) level of client burden; 3) quality of their relationship and interactions with the PA, 4) intervention participation levels; and 4) satisfaction with the intervention content and PA.

### Analysis

Descriptive statistics of participant demographic and background characteristics (from the baseline telephone interview), and the risk factor distribution (as identified on the ACASI screener) were computed. Means, standard deviations, and percentages were also calculated to assess intervention implementation using full study data collected through the centralized data monitoring system; statistics calculated included the number of intervention sessions attended, the mean length of interventions sessions stratified by the number of risk factor content areas covered, and implementation fidelity (as measured by the number of intervention sessions where all required risk factor content was fully or partially covered). Descriptive statistics were also calculated for the debriefing subsample of women to assess women' perceptions of study participation and satisfaction with the level of care received (for women in both groups), and acceptability and usefulness of the intervention overall, and the content related to each risk factor (for women in the intervention group only). Chi-square test statistics and analysis of variance procedures were used for all comparisons between the intervention and usual care groups. Between group comparisons were made in demographic characteristics, the distribution of identified risk factors, and in relation to perceptions of study participation for the subsample. A qualitative description of the obstacles encountered during program implementation and participant safety and adverse events is also provided.

## Results

### Participant Characteristics and Risk Distribution

Of the 1,070 women successfully enrolled in DC-HOPE, a small number of other minorities were enrolled and randomized, but only the 1,044 Black/African American women are included in this paper. The distribution of risk factors among the enrolled African American study participants is shown in Table 1.

**Table 1:** Risk factor distribution among African American women enrolled in the intervention study (N = 1044)

**Characteristic**	**N**	**%**
**Individual Risk Factors**		
Smoking Risk	500	47.9
ETSE Risk (Non-Smokers)	459	44.0
Depressive Symptoms Risk	373	35.7
Partner Violence Risk	216	20.7
		
Single vs. Multiple Risk Factors *		
Single Risk Factor	636	61.0
Two Risk Factors	312	29.8
Three Risk Factors	96	9.2
		
Risk Factor Overlap *		
Smoking Only	567	54.3
*Smoking (with or without ETSE)*	270	25.9
*ETSE Only (non-smokers)*	297	28.4
Depressive Symptoms Only	59	5.7
Intimate Partner Violence Only	10	1.0
Smoking and Depressive Symptoms	202	19.3
*Smoking and Depressive Symptoms*	116	11.1
*ETSE Only and Depressive Symptoms*	86	8.2
Smoking and Intimate Partner Violence	94	9.0
*Smoking and Intimate Partner Violence*	50	4.8
*ETSE Only and Intimate Partner Violence*	44	4.2
Depressive Symptoms and Intimate Partner Violence	16	1.5
All Three Risks	96	9.2
*Smoking, Depressive Symptoms, IPV*	64	6.1
*ETSE Only, Depressive Symptoms, IPV*	32	3.1

* Only three possible risk factor combinations are presented since women with any tobacco risk received intervention content depending on their risk status; either as smokers (defined by whether they smoked within 6 months or since becoming pregnant) or as non-smokers who were exposed to environmental tobacco smoke.

Ninety-two percent of women screened in with tobacco risk (either as active smokers or having ETSE), followed by depressive symptoms (36%), and IPV risk (21%). Sixty-one percent screened in with only one risk factor (tobacco, depressive symptoms, or IPV), 30% had two, and 9% had all three risks. There was considerable risk overlap among risks; the highest being for smoking and depressive symptoms (with or without IPV).

Women who participated in the study were, on average, 25 years old and were at 19 weeks of gestation at the time of the baseline interview. The majority was single, separated, widowed or divorced (76%). Over half of the women had other children (68%), were not working (64%), and received Medicaid (78%). Table 2 provides demographic characteristics of the study population as reported on the baseline interview. As a result of successful randomization, no differences were found for maternal characteristics or risk factor distribution between intervention and usual care groups.

**Table 2:** Demographic Characteristics of Study Participants

**Characteristic**	**Total (N = 1044)**	**Intervention (N = 521)**	**Usual Care (N = 523)**
	**Mean ± SD**	**Mean ± SD**	**Mean ± SD**

Maternal age (years), mean	24.6 ± 5.4	24.4 ± 5.5	24.8 ± 5.3
Gestational Age at baseline (weeks), mean	18.9 ± 6.9	19.3 ± 7.0	18.6 ± 6.8

	**N (%)**	**N (%)**	**N (%)**

First birth for mother	335 (32.1%)	172 (33.1%)	163 (31.2%)
Education level			
< High school	316 (30.3%)	159 (30.5%)	157 (30.0%)
HS graduate/GED	486 (46.6%)	245 (47.0%)	241 (46.1%)
At least some college	242 (23.2%)	117 (22.5%)	125 (23.9%)
Relationship status			
Single/separated/widowed/divorced	797 (76.3%)	396 (76.0%)	401 (76.7%)
Married or living with partner	247 (23.7%)	125 (24.0%)	122 (23.3%)
Working	381 (36.5%)	185 (35.5%)	196 (37.5%)
Receives Medicaid	810 (78.0%)	409 (79.1%)	401 (76.8%)

Note: Percentages are shown in parentheses.

As reported elsewhere [99], a total of 849 women completed the study, for a retention rate of 79%; five percent dropped out and 12% were lost-to-follow up. Women retained in the study and those not retained were not statistically different with regard to most sociodemographic characteristics and the targeted risk factors [99], however, women retained were slightly more likely to be married or living with a partner than those not retained (25% vs. 17%; p < .05).

### Intervention Participation and Implementation

#### Intervention Attendance and Visit Characteristics for All Intervention Women

The 521 women randomized to the intervention group attended a total of 2,417 intervention sessions. On average, each participant attended 4.6 intervention sessions out of a possible 10 recommended sessions. Four sessions, hypothetically, was considered a minimum adequate exposure to the intervention. As shown in Table 3, 85 women (16%) did not attend any sessions (but were retained in the study if they agreed to complete the telephone evaluations), while 313 (60%) attended four sessions or more. The average length of an intervention session was 35.6 minutes overall. Consistent with most women having a single risk factor, one targeted risk topic was covered in the majority of sessions. The length of sessions covering one risk lasted on average 31 minutes, and increased with the number of risk topics covered to 45 and 55 minutes respectively when two and three risks were covered. All sessions included general and reproductive behavioral health risk content totaling no more than 5 minutes. In sessions where "0" or no risk content was covered, as happened in 84 sessions (4%), either the participant refused to discuss the targeted risk factor content, or an urgent issue took precedence over the targeted risk topics.

**Table 3:** Intervention Session Attendance and Length of Sessions by the Number of Risk Factor Topics Covered

**Characteristic**	**N**	**%**
**Number of Prenatal & Postpartum Intervention Sessions Attended**		
None	85	16.3
1 – 3 Sessions	123	23.6
4 – 6 Sessions	144	27.6
7 – 9 Sessions	129	24.8
10 or more Sessions	40	7.7
**Total Number of Women in Intervention Group**	**521**	**100.0**

	N *	Mean ** ± SD

Length of Intervention Sessions by the Number of Targeted Risk Factor Topics Covered ***		
0 risk factor topics covered ****	84	13.2 ± 8.5
1 risk factor topic covered	1458	30.7 ± 10.1
2 risk factor topics covered	654	44.7 ± 13.3
3 risk factor topics covered	166	54.4 ± 14.3
**Total Number of Sessions Attended**	**2362**	**35.6 ± 14.5**

* There was incomplete data for 55 of the n = 2417 sessions: 22 without information on risk topics covered, and 33 additional sessions with incomplete data on session length.

** Average length of time per session is reported in minutes and includes all content covered: general, one or more of the three designated risk factor topics & reproductive risk coverage.

*** The maximum number of targeted risk factor topics that could be covered in any session was three: depressive symptoms, IPV, and tobacco risk (for smokers vs. non-smokers).

**** For sessions where "0" risk factor topics were covered, either the participant refused to discuss that content or an urgent issue took precedence over the targeted risk topics.

Women assigned to the usual care group met with their primary care providers as per standard clinic practice.

#### Implementation Fidelity of Pregnancy Advisors for All Intervention Sessions

Data on delivery of required risk factor content, as self-reported by the PAs, for the intervention sessions that participants attended were assessed. On average, 77% of sessions were completed as prescribed; that is, where all risk factor content was "fully" covered (data not shown). As can be seen in Table 4, the required general and risk specific intervention content was "fully" or "partially" covered in over 80% of the sessions attended for nearly all of the risk factor content areas. Approximately 10% of the sessions had to be abbreviated. For 32% of intervention sessions, there was some type of interruption.

**Table 4:** Number and Percentage of Sessions Attended Where the Pregnancy Advisors Covered Required Risk Factor Content *

	**General Content (N = 2417)**		**Depressive Symptoms Risk (N = 1019)**		**IPV Risk (N = 539)**		**Active Smoking Risk (N = 1175)**		**ETSE Risk (N = 1068)**		**Reproductive Risk (N = 2417)**	
	**n**	**%**	**n**	**%**	**n**	**%**	**n**	**%**	**n**	**%**	**n**	**%**

Fully Completed	2085	86.3	858	84.2	338	62.7	838	71.3	846	79.2	2064	85.4
												
Partially Completed Content	12	0.5	28	2.8	31	5.8	64	5.5	49	4.6	23	1.0
Omitted Content**	3	0.1	47	4.6	53	9.8	62	5.3	41	3.8	179	7.4
												
Participant Refused Content**	0	0.0	5	0.5	66	12.2	55	4.7	50	4.7	5	0.2
												
Marked Content N/A	196	8.1	40	3.9	22	4.1	91	7.7	29	2.7	10	0.4
Result Not Documented	121	5.0	41	4.0	29	5.4	65	5.5	53	5.0	136	5.6

NOTE: Percentages may not add to 100% due to rounding.

* Pregnancy advisors reported at each session whether the topic was covered "fully" (meaning they completed the entire script and slides for that risk factor area), "partially" (meaning they were only able to complete part of the content), and if they were not able to complete the content at all, they indicated the reasons for non-completion.

** PA's were instructed to omit content if a woman refused or expressed strong resistance, and for women who refused to discuss smoking cessation or reduction, to cover ETSE content instead.

The IPV and active smoking risk factor components were the areas of intervention presentation that were most often incomplete. The IPV intervention content was least likely to be covered fully/partially (in only 68% of the sessions), followed by the active smoking risk factor (in only 77% of sessions). PAs were instructed to omit content if women refused or expressed strong resistance, which happened most frequently in the IPV (22%) and active smoking risk sessions (10%), and for active smoking, to cover passive smoking instead. In 36% of the sessions where active smoke content was omitted or where women showed resistance or refused (n = 117 sessions), the PAs addressed ETSE content instead (data not shown).

### Overall Study Experiences and Challenges

#### Risk Reassessments for All Intervention Women

Periodic risk reassessments at 8 week intervals were conducted by the PA with intervention women to identify any new risks that the participants had developed or were willing to acknowledge since the original screening. In all, 68 women (13%) added a new risk during the course of the intervention. Depression was the most commonly acknowledged risk on reassessment, with 50 women being identified as having depressive symptoms. Twelve women were subsequently identified as being at risk for intimate partner violence, seven women were identified as being at risk for active smoking, and three women were identified as being at risk for passive smoking through these reassessments (note that some women may have added more than one risk so are represented in more than one category of newly identified risk).

#### Obstacles Encountered During Intervention Implementation

Increasing access to needed mental and behavioral health services through co-location within a primary healthcare setting was a goal of the study. About 80% of the sample attended at least one intervention session, and 60% completed at least 4 sessions. Relationships of counseling staff with the health providers in the clinics were generally good. Every attempt was made not to have intervention sessions interfere with clinic procedures and flow. Research team members participated in staff meetings at each clinic site prior to implementation at that site. Clinic staff was oriented to the goals and procedures of the study and their questions answered. The area of greatest confusion for the clinic personnel was in understanding the random assignment of the study design. Staff often wished to refer patients to the intervention and often assumed if a woman were participating in the study, that she was receiving additional services. Because the clinic staff members were not informed of a patient's study arm assignment, the research team reminded them that they must continue providing the guidance and education to all women, that they usually do during prenatal care.

While the PAs' complete delivery of content for specific risk related intervention components was successful in the majority of sessions, some obstacles were encountered that affected content presentation. Even when women attended the intervention sessions, and some of the topics were covered, the PAs were not always able to adequately address all of the risk components for women with multiple risks. Problems in delivery of the full content occurred for a number of reasons. Approximately 10% of the sessions had to be abbreviated (e.g., because women had to leave or the PA had conflicting appointments with other participants). For 32% of intervention sessions, there were other distractions or interruptions (e.g., women had to return to complete a clinic procedure, or a child accompanying the mother was disruptive) that may have interfered with intervention delivery as well as participant receipt of the material.

Intervention adjustments had to be made to both the IPV and the active smoking intervention components after the first few months in the field due to the difficulties encountered in delivery of that content for some women. Rather than risk increased drop-out rates from forced discussions of topics that might be unwanted or uncomfortable for participants to address, we elected to adjust the requirements for intervention coverage in these two areas. For both components, the PAs were instructed to make every effort to complete the content as it was originally designed for all women at risk. If a woman simply did not want to discuss their experiences or this issue any further, or if the PA felt that the relationship might be jeopardized, they were instructed to alter the delivery. For the IPV component, the PAs were instructed to omit the content entirely if it became too sensitive for women to discuss. For the active smoking component, which focused on both smoking cessation and significant reduction, the PAs were instructed to pursue a harm reduction strategy, and to cover the ETSE prevention topics instead. Decisions to omit the IPV or the smoking risk factor content were always made in consultation with intervention supervisors. The door was always left open to women for further discussions of IPV and smoking cessation or reduction, if and when women felt ready.

#### Participant Safety and Adverse Events During Implementation

Safety and well-being of the participants was of primary importance in the study. Women identified by study personnel as being in need of immediate medical, psychiatric, or police services were brought to the attention of primary care staff in the clinic following the specific referral procedures of that particular site. Involuntary termination of a woman's pregnancy (miscarriage) and other fetal loss (e.g. stillbirth), (primary outcomes of importance to the main study) were reported as adverse events, although participation in the intervention study was never considered to be a causal factor by the Data Safety Monitoring Board. The nature of the study population also presented a potential for other types of adverse events. With depression and/or partner abuse included among the targeted risks, it was recognized that decline in mental health status and episodes of partner violence were a very real possibility over the course of some women's participation. No causal relationship with the intervention was expected and none was identified. Disclosure of suicidal ideation in response to a question on the A-CASI screening was an exclusionary criterion for enrollment. Women who responded on screening that they would like to kill themselves, would kill themselves if they had a chance, or had a plan for taking their own life were immediately excluded from the study. Since screening was administered in the OB/GYN clinic, women who reported suicidal thoughts at that time were immediately identified to the clinical care provider designated by the site for this purpose. The number of women who reported suicidal ideation at screening was small [N = 2]. Suicidal ideation disclosed by enrolled intervention women to a PA was reported to the study's intervention supervisor and to the designated clinic staff member; the latter being responsible for referral. This occurred in two instances at an initial intervention session, in two women during the course of intervention and in two women in the postpartum period (one of whom had not attended any prenatal intervention sessions). Suicidal thoughts were disclosed during the phone evaluations in 13 baseline, five prenatal follow-up and 10 postpartum interviews. Women who responded during the phone interview as being distressed at least moderately by thoughts of ending their lives, a question on the Hopkins Symptom Checklist-Depression Scale (the depressive symptom scale used on the repeated measure evaluation battery) were immediately referred by the telephone interviewer to the mental health team of psychiatrists and clinical psychologists, who were on call to respond to these incidents. These consultants followed up by phone with participants, whether in the intervention group or identified by telephone interview, for whom there was concern. The woman's level of suicidal risk was determined, and subsequent contact made to the designated clinic staff person to initiate referral or, in one instance, contact emergency services to intervene. If women were considered to be suicidal, they were discontinued from the study after notification to the medical provider and provision of appropriate psychiatric referral. During the course of the study, eight enrolled women were excluded because of suicide risk. There were two reported incidents of contemporaneous intimate partner violence involving enrolled woman resulting in brief hospitalizations for evaluation of any effects on the pregnancy. At the time of disclosure, one incident had already been reported by the woman to police and medical personnel, and in the other, the woman chose not to take any actions. While there is no mandatory reporting requirement for IPV in Washington DC, the women were encouraged to discuss the issues with their doctors and the clinic social work staff. Contact information was provided for domestic violence hotlines and for emergency shelter should these be needed. These incidents were filed with the Institutional Review Board as adverse events.

### Debriefing Subsample Findings

#### Overall Perceptions and Satisfaction with Study Participation and Services

A 20% subsample (n = 183) of women were randomly identified to complete a postpartum telephone debriefing interview after completion of their participation in the study. Eighty-three percent (n = 152) of this group were able to be contacted by phone and completed the interview. No significant differences were found between intervention and usual care women in debriefing completion or refusal rates.

As can be seen in Table 5, the risk factor distribution for women in the subsample was similar to the distribution of risk factors for the full sample of women displayed in Table 1. Both the intervention and usual care women were quite positive about their overall participation and experiences during the DC-HOPE study; both groups thought the DC-HOPE recruitment (88% overall) and telephone interview staff (91% overall) were respectful and courteous, felt the pre-enrollment information they received about study participation was good (92% overall), and were satisfied with DC-HOPE study participation in general (93% overall). Women in both groups were also interviewed about their general prenatal care experiences. Most looked forward to their prenatal care (PNC) medical visits (76% overall), found the quality of PNC information and services they received to be helpful and good (67% overall), and were satisfied with their prenatal care services (63% overall). No significant differences existed between intervention and usual care women in this regard.

**Table 5:** Debriefing Subsample Participants: Risk Factors and Perceptions of Study Participation by Group

**Characteristic**	**Intervention(N = 78)**		**Usual Care(N = 74)**		**Overall(N = 152)**	
	**n**	**%**	**n**	**%**	**n**	**%**

Individual Risk Factor Distribution*						
Depressive Symptoms	26	33.3	25	33.8	51	33.6
Intimate Partner Violence	14	17.9	14	18.9	28	18.4
Active Smoking	33	42.3	36	48.6	69	45.4
ETSE Only (non-smokers)	40	51.3	35	47.3	75	49.3
						
**Positive About Experiences & Relationship with DC HOPE Staff ("Good")**						
Recruitment Specialist*	68	87.2	65	87.8	133	87.5
Telephone Interviewer*	72	92.3	67	90.5	139	91.4
Pregnancy Advisor **	57	93.4		NA		NA
						
**Looked Forward to Visit ("Very Much" or Quite a Bit")**						
Prenatal Care*	60	76.9	58	78.4	118	77.6
Pregnancy Advisor **	50	82.0		NA		NA
						
**Helpfulness of Information and Services Received (% reporting "Very Helpful")**						
Prenatal Care*	53	68.8	48	64.9	101	66.9
Computer slide supports **	49	80.3		NA		NA
DC Hope brochures and handouts **	50	82.0		NA		NA
Completing homework assignments **	47	77.0		NA		NA
Talking with Pregnancy Advisor **	60	98.4		NA		NA
						
**Quality of Information/Services Received ("Good")**						
DC Hope Project Information *	72	92.3	68	91.9	140	92.1
DC Hope Pregnancy Advisor Services **	51	83.6		NA		NA
						
**Satisfaction with Participation in DC-HOPE & Level of Care Provided (% reporting "Very Satisfied")**						
DC Hope Project (Participation in Study) *	71	91.0	70	94.6	141	92.8
Prenatal Care Clinic Staff (Level of Care from)	52	66.7	44	59.5	96	63.2
Pregnancy Advisor (Level of Care from) **	57	93.4		NA		NA

* Questions asked of both the intervention and the usual care group women. No statistically significant group differences were found.

** Questions asked of intervention group women. The denominator for Intervention group only questions was n = 61.

Women in the intervention group were quite positive about their relationship with the PA. The overwhelming majority found talking to the PA to be very helpful (98%), were satisfied with the level of care they received from the PA (93%), and looked forward to their visits together (82%). Over 80% of intervention women perceived the educational materials and the quality of the services they received to be "very helpful" or "good" (e.g., 82% found the brochures and materials and 80% found the slides to be helpful). Slightly fewer women found the homework assignments to be helpful (77%). In sum, the majority seemed to find the intervention experience rewarding and very useful. Where comparable questions were asked about PNC experiences (e.g., looking forward to the visit, and satisfaction with level of care), the DC-HOPE information and services were rated favorably by more intervention women than was their prenatal care.

#### Subsample Participation in the Intervention

Despite these data, a number of women in the debriefing subsample, as in the larger study sample (in Table 3), did not attend all of the intervention sessions. The distribution of the debriefing subsample in terms of their intervention attendance, and their reasons for not attending any or fewer sessions are presented in Table 6.

**Table 6:** Debriefing Subsample Participants: Number and Percentage of Intervention Group Sessions Attended & Reasons for Non-Attendance

**Characteristic**	**N**		**%**	
**Number of Sessions Attended**				
None	17		21.8	
1–3	18		23.1	
≥ 4 sessions	43		55.1	
**Total**	**78**		**100.0**	

	**Attended No Sessions(N = 17)**		**Attended 1–3 Sessions(N = 18)**	

	**n**	**%**	**n**	**%**

**Reasons for Not Attending Sessions**				
Not informed/advised to come back	14	41.2	2	5.6
Not interested/does not need risk topics	10	29.4	2	5.6
No time/sessions too long	8	23.5	6	16.7
Moved from area	4	11.8	4	11.1
No Comment/Not sure	2	5.9	8	22.2
Miscarriage	2	5.9	0	0.0
Reproductive Health Reasons (e.g., premature delivery, late PN care entry, stopped after delivery).	0	0.0	6	16.7
Only attended when had prenatal care	0	0.0	6	16.7
Knew information already	0	0.0	4	11.1
Convenience; location	0	0.0	4	11.1
Sick, in hospital during pregnancy, not feeling well	0	0.0	2	5.6

Perhaps most important among these data were women's reasons for non-participation, which are presented separately for the seventeen women who did not attend any of the sessions and for the 18 women who attended at least one, but less than the minimum number established (1–3 sessions). Top reasons given by women who did not attend any sessions were that the PA had not informed or advised them to come back (41%), they did not feel they needed/wanted to participate in the intervention (29%), and the sessions were too long (24%). Women who attended 1–3 sessions reported they were not sure or did not know why (22%), similarly reported that sessions were too long (17%), and/or enrolled late in pregnancy, delivered early, stopped attending after giving birth, or only participated when they went to the prenatal care clinic (17%).

#### Subsample Intervention Participant Perceptions and Satisfaction with Each Risk Factor Component

The perceived helpfulness of the various risk factor session content is shown in Table 7, along with perceptions of the likelihood of continuing to use the information and skills received, for all intervention women in the debriefing subsample who attended at least one intervention session (n = 61), and for women presenting with multiple risk factors. Most intervention women reported that the information and skills they received for each specific risk factor was "very helpful", and despite the small numbers of women, there were several significant differences in the perceived helpfulness of each risk factor component. The active smoking, ETSE and reproductive health risk factor intervention components were perceived by more women as being helpful than the psychosocial intervention components (i.e., depression and IPV). Significant differences were found in Chi-square test comparisons between the perceived helpfulness of the reproductive health and the depression or intimate partner violence (*p *≤ .01 for both comparisons), between the ETSE and the depression (*p *≤ .01) or intimate partner violence (*p *≤ .05) content, and between the active smoking and the depression (*p *≤ .01), but not the smoking or intimate partner violence content.

**Table 7:** Debriefing Subsample Participants: Perceptions of Target Risk Factor Content Among Women Attending Any Intervention Sessions.

**Characteristic**	**Depression**		**Intimate Partner Violence**		**Active Smoke**		**Passive Smoke Only**		**Passive Smoke: All Others**^a^		**Repro-ductive Risk**^b^	
**# Women Identified to Receive Risk Factor Content**	**(N = 26)**		**(N = 14)**		**(N = 33)**		**(N = 40)**		**(N = 38)**		**(N = 78)**	

**Intervention Session Attendance**^c^	**n**	**%**	**n**	**%**	**n**	**%**	**n**	**%**	**n**	**%**	**n**	**%**
Women with Risk Factor Content Who Attended ≥ 1 Session	20	76.9	11	78.6	26	78.8	31	77.5	30	78.9	61	78.2

**# Women Attending ≥ 1 Session**	**(N = 20)**		**(N = 11)**		**(N = 26)**		**(N = 31)**		**(N = 30)**		**(N = 61)**	

**Perceptions of Information & Skills Covered **^d^	**n**	**%**	**n**	**%**	**n**	**%**	**n**	**%**	**n**	**%**	**n**	**%**
Information & Skills Were "Very" Helpful "Very" Likely to Use Information & Skills in Future:	15	75.0	8	72.7	24	92.3	28	90.3	27	90.0	54	88.5
To prevent each target risk factor	12	60.0	10	90.9	27	65.4	27	87.1	23	76.7	--	--
To prevent getting a sexually transmitted disease.	--	--	--	--	--	--	--	--	--	--	55	90.2
To prevent getting pregnant again too soon.	--	--	--	--	--	--	--	--	--	--	51	83.6

**# Women With Multiple Risk Factors**			**(N = 5)**		**(N = 7)**^f^		**(N = 9)**		**(N = 11)**^f^			

**Helpfulness of Multiple Risk Factor Content **^e^	**n**	**%**	**n**	**%**	**n**	**%**	**n**	**%**	**n**	**%**	**n**	**%**
Depression Content "Helpful" in Reducing Other Risks	--	--	--	--	4	57.1	7	77.8	6	54.5	--	--
IPV Content "Helpful" in Reducing Other Risks ^f^	--	--	3	60.0	1	50.0	7	77.8	1	50.0	--	--

^a ^At the postpartum visit, all intervention women received information about environmental tobacco smoke exposure risks for their new baby irrespective of risk levels. This column represents all women who were not in the passive smoke only intervention group.

^b ^All intervention group women were provided reproductive risk information irrespective of risk levels.

^c ^Extent women who were required to receive risk factor content attended ≥ 1 intervention session.

^d ^Extent women with each risk factor perceived the intervention information and skills covered as being "Very" helpful or who reported they would be "Very" Likely to continue to use the skills and information in the future.

^e ^Extent women with multiple risk factors who perceived that receiving the depression or IPV content helped them to reduce the other target risk factors "Quite a bit."

^f ^For women with multiple risk factors who received both the IPV content and the "Active Smoke" or "Passive Smoke: All Others" content, the N = 2.

Fewer intervention women indicated that in the future they would be "very likely" to use the information and skills they received than found the sessions helpful, except in relation to the IPV intervention component, for which more women indicated they would be "very likely" to use the information/skills in the future than felt the intervention was "very helpful" (91% vs. 73%). Women responded to the reproductive health content by expressing a significantly greater likelihood of using the information they received to prevent future STIs than to prevent pregnancy (90% vs. 84%; Chi-square = 48.12, df = 6; *p *≤ .001). More women at risk for ETSE than those with active smoking risk in conjunction with another risk factor, found the receipt of the depression or the intimate partner violence content helpful to them in reducing environmental tobacco smoke exposure.

## Discussion

Despite interest in developing effective approaches to identify and intervene on multiple adverse health behaviors, little is known about the most effective ways to reduce complex risk [4,5,111]. In this paper, we describe one intervention approach that may serve to increase understanding of necessary considerations in attempts to address psychosocial and behavioral risk factors presented by pregnant women in prenatal care settings. Previous investigators have also cited important issues to consider when planning and implementing such an approach [4,5]. While this intervention study was not designed to empirically test or answer these questions directly, we describe the decisions that went into the selection of multiple risk behaviors, the conceptual framework applied, and theoretical constructs and models thought to be common or unique across behaviors that could be addressed simultaneously in conjunction with prenatal care visits. We also provide important information on intervention adherence and implementation fidelity, and describe the challenges faced during implementation.

This study demonstrates the feasibility of incorporating a computerized screening procedure for psychosocial and behavioral risk factors into prenatal care settings serving low income African American women. One of the acknowledged problems in addressing needs of underserved populations is the failure of primary care providers to identify psychosocial health risks despite recommendation by American College of Obstetrics and Gynecology [112,113]. The use of the ACASI screening technique in this study was well accepted by the study population, provided a sense of confidentiality for their responses and minimized administrative time for clinic personnel. Screening resulted in the identification of the pregnancy risks of smoking, ETSE, depressive symptoms and intimate partner violence that were present in a substantial portion of this urban African American population. Re-screening at later points during the pregnancy may also be warranted because additional risks were declared following initial screening in this study. Subsequent reporting of risks, either through formal reassessment or in discussion with PAs or phone interviewers, may have been due to greater openness once a relationship developed with the counselor or other study staff, because of new risk development over the course of pregnancy, or within subject variations in risk factors across trimesters as has been found elsewhere for cigarette smoking and other substance use [114,115].

Despite the need, at times, to remain beyond the completion of the prenatal clinic visit to participate in the intervention, 60% of the intervention group completed at least 4 of the recommended 10 intervention sessions. This contrasts with the less than 50% percent of women in primary care with mental health problems that pursue recommended mental health services when they are not co-located within a primary care setting [116]. However, willingness to participate in sessions was variable across the group. Some women found it difficult to remain for a longer time to complete the intervention after their medical visit. As reported anecdotally by the PAs, some participants avoided the PA when she attempted personal contact with them or did not appear for their intervention session despite completing the medical visit. As noted by Klinkman [117], in contrast to patients presenting in mental health settings, patients in primary care may not acknowledge psychosocial or behavioral problems and may not want or expect intervention. This was particularly evident with the smoking cessation and the intimate partner violence interventions, where some women were not prepared to discuss these issues and adjustments had to be made to omit certain content when women refused, or switch to passive smoke exposure prevention if women were unwilling to discuss cessation or reduction. In such cases, acceptance of the problem and motivation for treatment may be difficult to achieve.

The necessary time limitations of the intervention sessions may present a challenge to providing adequate intervention exposure for women with multiple risks. For the majority (61%) of women with a single risk, the sessions were a little over a half hour on average. When women had 2 or more risks, the sessions lengthened considerably to between 45 and 55 minutes. Making intervention sessions too long would put an unrealistic time burden on women already making time for the prenatal care visit, and possibly defeat the advantage of co-locating behavioral intervention in a primary medical care setting. A greater impediment to intervention exposure was the inconsistency in adherence to prenatal health visits, as is common in women with psychosocial and behavioral risks [118]. When a patient skipped a monthly scheduled prenatal medical visit, despite phone reminders and encouragement by the PA to reschedule the visit, the intervention sessions might be at least 8 weeks apart, resulting in lack of continuity in intervention delivery.

Of the subgroup sampled for satisfaction with their participation, over 90% in both the intervention and usual care groups rated their study experiences positively. This may reflect the success of recruiters and follow up staff in maintaining positive interactions in completing their tasks with participants. Monetary incentives may also have contributed to women feeling adequately compensated for their participation. Intervention acceptability was also high among women in the intervention group, perhaps because of the focus on multiple vs. single risk factors or the flexibility and sensitivity demonstrated in our approach. Ninety-eight percent of women found talking with the PA, and over 80% found the intervention materials, to be helpful. Anecdotally, women reported that it was nice to have someone to talk to, and who expressed concerns about their thoughts and feelings. This was reinforced by the fact that women reported satisfaction with the intervention at a higher level than they did their prenatal care experience.

Women receiving the intervention for active smoking and ETSE acknowledged that the intervention was relevant to their needs. Because of the many public health warnings about smoking during pregnancy, women who are smokers may recognize the importance to their infants of the smoke reduction strategies that were offered in the intervention. Women with ETSE risk, or with IPV risk in the debriefing subsample, were more likely to report that in the future they would continue to use the information and skills that they learned through the intervention. Both of these interventions focused on harm reduction strategies (i.e., removing oneself from high risk situations). These are perhaps easier skills to continue to use than ones needing more active changes in behavior, as is required in smoking cessation or depression reduction.

A possible limitation to the debriefing subsample results include the fact that the sampling to identify this 20% subgroup of women was not stratified by target risk factor, but rather by intervention and usual care groups. This limited our understanding of women's experiences with specific risk factor content, and made understanding of the benefits of addressing multiple risks impossible. Sampling a broader segment of the study population, or limiting the debriefing interviews only to women who participated in the intervention arm, or over-sampling of smaller groups would have increased these numbers, and facilitated our understanding of the acceptability and perceived benefit of each risk factor component. Although the full study data do not include participant perceptions of the intervention, some of these issues, and potentially our proposed conceptual framework which addresses multiple psychosocial and behavioral risks simultaneously, may be able to be further explored with the process, impact and outcome data from the full sample. Evidence for the synergistic effects of covering multiple risk behaviors and psychosocial factors as was proposed in the conceptual model are not possible to address in this paper, given the limited numbers of women in this subsample with multiple risks.

The generalizability of these findings are obviously limited to lower income, urban, and pregnant African American women over 18 years of age who seek prenatal care services.

In a population dealing with many competing stresses, difficulties remain in enhancing motivation for change and with treatment adherence. The results of the current study with about 60% of the sample completing a minimum exposure to the treatment are more favorable than that of Brown et al [119] who found that only 45% of African American primary care patients pursued adequate follow-up for recommended psychotherapy. However, despite making every effort to have the DC-HOPE intervention be as convenient as possible for women attending primary care visits, a substantial minority of the sample attended fewer than four sessions. A challenge remains in the need for brief intervention models with clear benefit as perceived by urban, low income minority populations.

One important factor in enhancing patient adherence to treatment for psychosocial problems and the success of integrated care is close collaboration between the primary healthcare provider and the mental health specialist [120,121]. In order for the primary care provider to remain blind to the study arm assignment of the patient (and thus able to maintain usual care practices), collaboration with the intervention counselor was restricted. Had the primary providers been able to support the value of the intervention sessions for non-compliant patients, increased motivation and adherence to the intervention might have been achieved for some women. Researchers developing similar community-based intervention models might consider allowing the primary care provider to encourage their patients to participate in cases where there is poor adherence to recommended psychosocial or behavioral intervention.

Another important factor, pertinent to enhancing implementation fidelity and participant retention, was being sensitive and responsive to participant needs, and flexible in relation to intervention delivery. Reasons for women to refuse or express reluctance to discuss the IPV and smoking intervention content are not fully clear. The IPV content was originally designed to be delivered in a single session and perhaps only a few sessions were necessary to fully cover the content [80]. Continued presentation may have been viewed by the participants as redundant. Another reason could be that the IPV intervention was information-based rather than focusing on psychosocial or therapeutic issues, or on behavior change theories or strategies. For example, the IPV intervention was somewhat circumscribed (e.g., understand the cycle of violence, prepare for reoccurrences by developing a safety plan, recognize early precursors and indicators of escalation of IPV, and take self-protective actions), and did not focus on other related factors such as a woman's history of violence exposures, and intergenerational family violence issues. The content was also sensitive; many women may not have been ready to change, or they could have changed partners already, since receipt of this risk factor was based on prior year IPV (whether with a current or prior partner). With regard to the active smoking intervention component, women who were in the pre-contemplation stage – basically women who reported that they were not going to quit or reduce their cigarette smoking during pregnancy – were the most resistant to this intervention content. Omitting the IPV content or shifting from the active to the passive smoking content when women expressed reluctance potentially helped in their retention. Had we not made these adjustments, we suspect that adherence to the intervention content by the PAs would have been lower, and the reported drop-out rates higher.

Masters level personnel were selected to provide the integrated intervention, particularly because of the mental health issues presented by depressed women, but also because intervention delivery for the depression, smoking and ETSE intervention content all required familiarity with behavioral counseling and CBT approaches. Because of the very structured nature of the intervention curriculum, bachelors level individuals from disciplines such as nursing, psychology or community health, with training and experience in behavioral counseling may have adequate skills for fulfilling the counseling role for women with smoking risks. The original intervention models for smoking and IPV risks from which DC-HOPE was adapted did not require Master's level professionals, but they also were not being delivered using a CBT framework which was one of the adaptations made in DC-HOPE. Working with women with mental health issues and application of CBT methods, however, may require at least Master's preparation. Ready access to supervision and emergency mental health support would remain a helpful component for the counseling staff.

The manual developed for the intervention had very explicit content for each session in script form. The visual supports for the patient presented on the computer screen were contained in a CD-ROM, which each PA received along with the intervention manual. This allowed for consistency and fidelity in the delivery of the intervention. These materials were developed to be readily reproduced and not require extensive training for use, with the hope that this would facilitate later replication of the model in other primary care settings.

Strengths of this study are in its success in identifying implementation obstacles and describing strategies for overcoming the challenges faced in providing a multiple risk factor intervention in community-based OB/GYN settings serving low income minority women. We provided some modest data on the sample characteristics, intervention participation and implementation assessed through process evaluation procedures put in place by the data coordinating center, and as reported by women themselves. The 20% debriefing subsample was representative in relation to attendance and risk factor distributions, and provided information regarding how intervention and usual care women felt about study participation overall and enabled a determination of the acceptability of the intervention among women randomized to the intervention group.

## Conclusion

Significant numbers of low income African American women in prenatal care have psychosocial and behavioral risks that may impact on pregnancy. An integrated intervention addressing multiple risks simultaneously can be implemented in a prenatal, primary care clinic setting, although there were formidable challenges that required both skill and flexibility in delivery. Further research is needed to evaluate the effectiveness of community-based interventions such as DC-HOPE. More research is needed to better understand ways to improve patient adherence to recommended interventions, and to help some women with complex risk to see the intervention as relevant to their needs. Through programs such as the DC-HOPE project, it is anticipated that needs of underserved populations such as pregnant minority women living in poverty may be better served.

## Competing interests

The authors declare that they have no competing interests.

## Authors' contributions

KSK was involved in study design, intervention model development, development and oversight of the depression intervention, oversight of the full study intervention implementation and intervention staff, data analyses, manuscript development and final editing of this submission. SMB was involved in study design, intervention model development, development and oversight of the active and passive smoking interventions, development of the process, impact and outcome evaluation measures, manuscript development and final editing of this submission. RAM was involved in study design, intervention model development, development and oversight of the reproductive risk intervention, and manuscript development. PWS was involved in study design, intervention model development, development and oversight of the IPV intervention and oversight and manuscript development. DBW was involved in study design, intervention model development, development of the reproductive risk intervention, and manuscript development. MFR was involved in study design, intervention model development, development of the active and passive smoking interventions, and manuscript development. MR was involved in study design, repeated measures oversight and manuscript development. KBM was involved with model implementation, data coordination and management, and manuscript development and revision.

## Pre-publication history

The pre-publication history for this paper can be accessed here:


